# An Appeal for the Queen's

**Published:** 1910-11-19

**Authors:** 


### AN APPEAL FOR THE QUEEN'S.

*To the Editor of* The Hospital.

Sir,—I am anxious to bring to the notice of your readers a strong case for their generous assistance.

The Queen's Hospital for Children, Hackney Road, Bethnal Green, which King Edward, together with her Majesty Queen Alexandra, had arranged to visit last summer only a few weeks before his sudden fatal illness, is one of the largest institutions of its kind in the kingdom, and has one of the smallest endowments. With 134 beds and an out-patient record of 87,000 attendances annually, it has an annual expenditure of £12,000 and an income from investments of £320.

Surrounded by some of the poorest districts of London, it has done splendid work for the past forty-three years amongst the class of children that most need such assistance. Owing, no doubt, to the fact of its being little known outside of the poor districts of London, it does not receive that valuable assistance from legacies which other and better known institutions enjoy, and it is nearly always the case that for the year's income it is entirely dependent upon the strenuous exertions of its friends in the collection of funds.

In spite of financial difficulties, which of course give rise to heavy appeal expenses, and notwithstanding the adoption of the most modern and most efficient methods and appliances in every department, this hospital's cost per occupied bed has for many years been one of the lowest in London.

At the present time it needs immediate assistance. A bank overdraft in respect of maintenance, which stood at £1,640 last January, has now grown to over £3,250, and this, coupled with a mortgage of £5,250 at 4 per cent. interest, makes the position one of great difficulty.

I am taking the chair at a dinner at the Hôtel Cecil on the 23rd instant in support of a special appeal for £8,500 to free the institution from debt, and I confidently ask all your readers to contribute in a small or large way towards this excellent object.

The work is well done, it is economically done, and it is absolutely essential to the public welfare that it should be fully maintained.

I may mention that this was formerly the North-Eastern Hospital for Children, the new name having been adopted in 1908 with the approval of her Majesty Queen Alexandra and by special permission of his late Majesty Edward VII.

Contributions may be sent to me or to the Secretary, Mr. T. Glenton-Kerr, at the hospital, and cheques should be crossed "Barclay and Co., Ltd."—I am, Sir, your most obedient servant,

Bethnal Green, E., November 10.

Shaftesbury,
*President.*

